# Contrast-Enhanced Mammography in Breast Cancer Follow-Up: Diagnostic Value in Suspected Recurrence

**DOI:** 10.3390/jimaging11120435

**Published:** 2025-12-06

**Authors:** Claudio Ventura, Marco Fogante, Nicola Carboni, Silvia Gradassi Borgoforte, Barbara Franca Simonetti, Elisabetta Marconi, Giulio Argalia

**Affiliations:** SOD Radiologia Materno Infantile, Senologica, Cardiologica ed Ecografica Ambulatoriale, Azienda Ospedaliero Universitaria delle Marche, Via Conca 71, 60126 Ancona, Italy; claudioventura20@gmail.com (C.V.); nicola.carboni@ospedaliriuniti.marche.it (N.C.); silvia.borgofortegradassi@ospedaliriuniti.marche.it (S.G.B.); barbarafranca.simonetti@ospedaliriuniti.marche.it (B.F.S.); elisabetta.marconi@ospedaliriuniti.marche.it (E.M.); giulio.argalia@ospedaliriuniti.marche.it (G.A.)

**Keywords:** contrast-enhanced mammography, breast cancer, recurrence, surveillance, diagnostic accuracy, personal history of breast cancer

## Abstract

Women with a personal history of breast cancer (PHBC) are at increased risk of local recurrence or new primary tumors, which are often difficult to assess on conventional imaging because of postoperative changes. This prospective study aimed to evaluate the diagnostic performance of contrast-enhanced mammography (CEM) in women with PHBC presenting with suspicious findings on follow-up mammography or ultrasound. Sixty-two patients underwent CEM between December 2023 and June 2025. Lesions showing enhancement were biopsied, while non-enhancing ones were followed for stability. Histopathology served as the reference standard. Diagnostic performance was assessed using standard statistical methods, including sensitivity, specificity, Fisher’s exact test, and ROC analysis. Among 62 lesions, 34 were enhanced on CEM; 30 of these (88.2%) were malignant, whereas 25 of 28 non-enhancing lesions (89.3%) were benign (*p* < 0.001). CEM demonstrated a sensitivity of 90.9%, specificity of 86.2%, and diagnostic accuracy of 88.7%. Interobserver agreement was substantial (κ = 0.76, *p* < 0.001). Enhancement on recombined CEM images was strongly associated with malignancy. These findings confirm that CEM provides excellent diagnostic performance in the surveillance of women with PHBC, effectively distinguishing benign from malignant postoperative changes. CEM may serve as a practical and accessible alternative to magnetic resonance imaging, particularly in patients with contraindications or where it is unavailable.

## 1. Introduction

Surveillance breast imaging plays a critical role in women with a personal history of breast cancer (PHBC), as they face a higher risk of developing new in-breast malignancies, which tend to be detected later than those found in the general screening population. Timely detection of recurrent or new primary breast cancers contributes to better survival outcomes. Notably, the reported interval cancer rate is 3.6 per 1000 mammographic examinations in women with PHBC, compared with 1.4 per 1000 in women without such a history [1,2,3,4]. Annual mammography (MG) remains the standard surveillance tool for women with a PHBC. Supplemental ultrasound (US) can be employed to improve sensitivity, although its use is limited by low specificity [5,6,7]. Breast magnetic resonance imaging (MRI) offers higher cancer detection rates and reduces the incidence of interval cancers. MRI is generally reserved for selected groups, including women diagnosed before the age of 50, those with dense breast tissue, or individuals with genetic predisposition [8,9]. However, its high cost, limited availability, and current recommendations prevent its routine use in surveillance programs [10]. Contrast-enhanced mammography (CEM) has recently emerged as a promising alternative in both screening and diagnostic contexts, with sensitivity approaching that of MRI and comparable specificity, while being more accessible and less resource-demanding [11]. CEM combines standard digital mammography with the administration of iodinated contrast, producing low-energy images equivalent to conventional two-dimensional MG, along with recombined contrast-enhanced images that provide functional information on lesion vascularity and perfusion. The fields of CEM application include problem solving for inconclusive findings, preoperative staging, and monitoring neoadjuvant therapies [12,13]. In patients with a PHBC, postoperative architectural distortion and scar tissue can make image interpretation challenging, particularly in symptomatic cases [14]. Although ongoing studies are investigating the role of CEM in follow-up among women with a PHBC [15,16,17], its use in this setting remains controversial. The value of CEM as a routine surveillance tool has not yet been established, given the limited literature and lack of consensus, with some authors recommending its application only in carefully selected cases [18,19]. Based on these considerations, the aim of our study was to assess the diagnostic performance of CEM in women with PHBC undergoing oncological follow-up.

## 2. Materials and Methods

### 2.1. Study Population

This study was approved by our Ethics Committee (protocol identification number and approval date: 2771—15 December 2022), and written informed consent was obtained from all participants.

This prospective study enrolled patients with a PHBC who underwent CEM between December 2023 and June 2025 due to new findings on follow-up conventional imaging (MG and/or US) classified as Breast Imaging Reporting and Data System (BI-RADS) 3, 4, or 5. Inclusion criteria were new imaging findings representing the first suspicion of local recurrence. Exclusion criteria included prior total mastectomy, presence of breast implants, renal impairment, contrast media allergy and pregnancy or suspected pregnancy. For all enrolled patients, both clinical and anamnestic data were systematically collected.

### 2.2. CEM Examination and Analysis

All CEM examinations were performed on a dual-energy system (Pristina Bright, GE HealthCare, Chicago, IL, USA). After intravenous injection of a non-ionic iodinated contrast medium (iopamidol, 1.2 mL/kg at 3 mL/s) followed by a 20 mL saline flush at the same flow rate, paired low-energy (23–32 kVp) and high-energy (45–49 kVp) images were obtained in both craniocaudal and mediolateral oblique projections. Recombined images were automatically generated to highlight contrast-enhancing areas. Image acquisition started 2 min after contrast administration and was completed within 10 min.

Before analysis, all datasets were standardized in terms of size, orientation, and calibration. The total breast compression time for a single exposure varied according to breast composition and thickness, typically ranging from 2 to 20 s. The applied compression force was maintained between 5 and 20 daN. Radiologists manually outlined regions of interest to minimize the influence of artifacts and adjacent structures, enabling focused assessment of lesion morphology and enhancement. All CEM images were independently reviewed by two experienced breast radiologists (10 and 15 years of experience), blinded to each other’s assessments. In cases of disagreement, a third senior radiologist (20 years of experience) adjudicated the findings to reach a final consensus. At CEM, lesions that showed contrast enhancement or were classified as BI-RADS 4–5 were referred for biopsy and surgical excision, while non-enhancing lesions and those categorized as BI-RADS 3 were followed up with imaging after 6 months. Lesions that regressed or remained stable during follow-up were classified as benign, whereas those showing suspicious changes on MG or US were subsequently biopsied and surgically excised.

### 2.3. Histopathological Analysis

Pathological assessment was carried out on surgical specimens by a dedicated breast pathologist with 15 years of experience. The analysis included tumor size, histological type (ductal or lobular), and histological grade (G1–G3) according to the Nottingham modification of the Bloom–Richardson system (Elston–Ellis criteria). Lymphovascular invasion and immunophenotypic profile were also recorded. Receptor status for estrogen (ER), progesterone (PR), and human epidermal growth factor receptor 2 (HER2) was determined by immunohistochemistry (IHC). Nuclear staining of ≥10% of tumor cells was considered positive for ER and PR. HER2 expression was scored as 0, 1+, 2+, or 3+ by IHC; cases with a 2+ score underwent fluorescence in situ hybridization (FISH) to evaluate HER2 gene amplification. Tumors were classified as HER2-positive if they showed either IHC 3+ expression or gene amplification on FISH, and as HER2-negative in case of 0 or 1+ IHC scores. Based on ER, PR, HER2, and Ki-67 expression, breast cancers were categorized into four immunophenotypic subgroups: Luminal A (ER and/or PR positive, HER2 negative, Ki-67 < 20%); Luminal B (ER and/or PR positive, HER2 negative, Ki-67 ≥ 20%, or ER/PR positive with HER2 positivity irrespective of Ki-67); HER2-enriched (ER and PR negative, HER2 positive); and triple-negative (ER, PR, and HER2 all negative).

### 2.4. Statistical Analysis

Statistical analyses were performed using MedCalc version 14.8.1 (MedCalc Software bvba, Ostend, Belgium). Continuous variables were reported as mean ± standard deviation, and categorical variables as frequencies and percentages. Interobserver agreement between the two breast radiologists was measured using weighted Cohen’s kappa. The diagnostic performance of CEM was evaluated in terms of sensitivity, specificity, positive predictive value, negative predictive value, and accuracy by comparing CEM findings with histopathological results, while follow-up data were used as the reference standard for benign cases. The association between lesion enhancement on CEM and histopathological outcome was assessed using Fisher’s exact test. Receiver operating characteristic (ROC) analysis was also evaluated. A *p*-value < 0.05 was considered statistically significant.

## 3. Results

### 3.1. Study Population

Out of 80 patients initially assessed for eligibility, 18 were excluded. The final study population consisted of 62 patients with a mean age of 67.1 ± 12.0 years and a mean body mass index of 23.7 ± 8.1 kg/m^2^. The time from first diagnosis was <5 years in 17 patients (27.4%), 5–10 years in 24 patients (38.7%), and >10 years in 21 patients (33.9%). Regarding clinical presentation, locoregional pain was reported in 18 patients (29.0%). Table 1 summarizes these findings.

Before CEM, based on follow-up conventional imaging alone, 31/62 lesions (50.0%) were BI-RADS 3, 19/62 (30.6%) were BI-RADS 4, and 12/62 (19.4%) were BI-RADS 5. Lesions were more frequently located in the left breast (40, 65.0%) than in the right breast (22, 35.0%). Regarding lesion location, the majority were in the upper outer quadrant (UOQ, 28, 45.2%), followed by the lower inner quadrant (LIQ, 11, 17.7%), lower outer quadrant (LOQ, 9, 14.5%), upper inner quadrant (UIQ, 8, 12.9%), and retroareolar region (RA, 6, 9.7%) (Table 2).

### 3.2. CEM Examination and Analysis

The distribution of glandular patterns among the studied breasts was as follows: pattern A in 3 cases (4.8%), pattern B in 34 (54.8%), pattern C in 17 (27.4%), and pattern D in 8 (12.9%). Background parenchymal enhancement was minimal in 32 cases (51.6%), mild in 18 (29.0%), moderate in 9 (14.5%), and marked in 3 (4.8%). Regarding the enhancement pattern of the main lesions, 26 lesions (41.9%) presented as masses, 2 (3.2%) as non-mass enhancement, 6 (9.7%) as asymmetries, while no enhancement was observed in 28 cases (45.2%) (Table 3). Among the 62 evaluated cases, both radiologists provided concordant interpretations in 56 (90.3%). Cohen’s kappa analysis confirmed a substantial level of agreement (κ = 0.76; *p* < 0.001). All non-enhancing lesions were BI-RADS 3.

### 3.3. Histopathological Analysis

**Figure 1:**
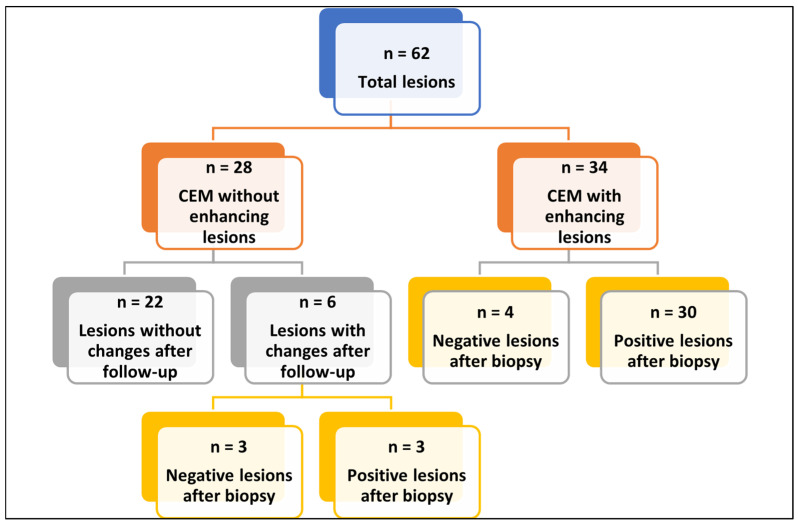
Flow-chart of CEM and histopathological analysis. Abbreviations—*n*: number of patients; CEM: contrast enhancement mammography. Flowchart summarizing the 62 breast lesions included in the analysis. Lesions were divided into those without enhancement on CEM (*n* = 28) and those with enhancement (*n* = 34). Among non-enhancing lesions, 22 remained stable at follow-up, while 6 showed changes and underwent biopsy, yielding 3 benign and 3 malignant results. Among enhancing lesions, biopsy revealed 4 benign and 30 malignant findings.

A total of 40 tumors were biopsied: 34 immediately after CEM and 6 after follow-up. Among the 40 tumors, 33 were malignant, and 7 were benign. Histological assessment revealed a clear predominance of infiltrating lesions (29 cases), represented by invasive ductal carcinoma (23 cases), followed by invasive lobular carcinoma (6 cases). Regarding immunophenotype, the most frequent subtypes were Luminal B (11 cases) and Luminal A (7 cases), whereas HER2-positive tumors were rare (2 cases). Most tumors showed a low-to-intermediate grade, with 17 cases classified as G1. Lymph node localization was in 2 cases (Table 4). Figure 1 shows the flow-chart of CEM and histopathological analysis.

### 3.4. Diagnostic Performance of Conventional Imaging and CEM

The diagnostic performance for conventional imaging was sensitivity of 81.8% (27/33), specificity of 86.2% (25/29), positive predictive value of 87.1% (25/32), negative predictive value of 80.6% (22/30) and accuracy of 83.9 (52/62%). Malignant lesions classified as BI-RADS 3 were 6/62 (9.7%). CEM demonstrated excellent diagnostic performance, with a sensitivity of 90.9% (30/33), specificity of 86.2% (25/29), positive predictive value of 88.2% (30/34), negative predictive value of 89.3% (25/28), and an overall diagnostic accuracy of 90.2% (55/62) (Table 5). Among the four enhancing but benign lesions, conventional imaging had originally classified them as BI-RADS 4. Among the three malignant but non-enhancing lesions, conventional imaging had classified all as BI-RADS 3, with none in the BI-RADS 4–5 categories. After CEM, all three malignant BI-RADS 3 lesions demonstrated clear enhancement and were correctly upgraded to BI-RADS 4–5.

**Figure 2:**
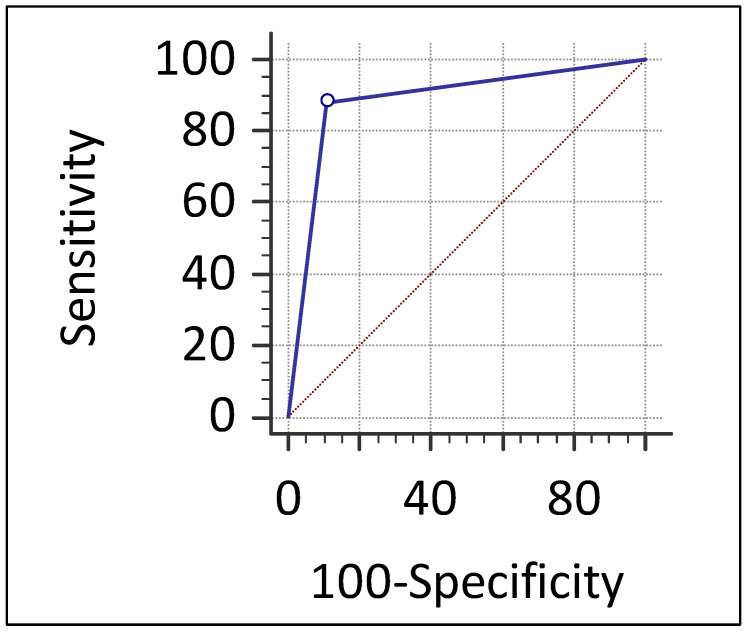
Receiver operating characteristic (ROC) curve of contrast-enhanced mammography (CEM) for detecting malignant lesions in the study cohort. The ROC curve of CEM shows excellent diagnostic performance for detecting malignant lesions, with an area under the curve (AUC) of 0.888 (95% CI: 0.782–0.954; *p* < 0.0001). The diagonal dashed line represents the line of no discrimination.

**Figure 3:**
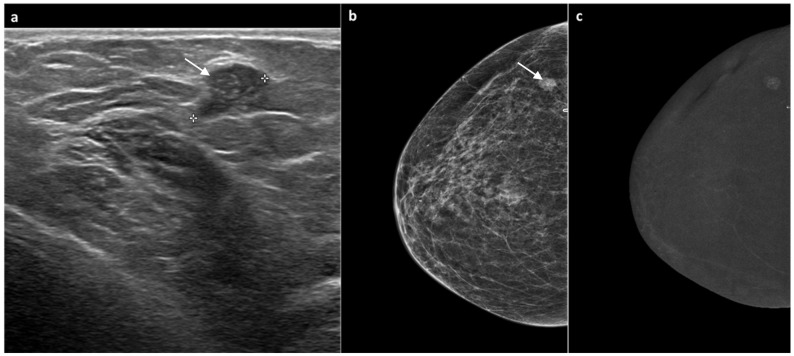
A 60-year-old woman, previously treated for ductal carcinoma in situ (DCIS) of the right breast with partial mastectomy six years earlier. Follow-up mammography showed a well-defined nodular opacity with associated microcalcifications adjacent to the surgical scar. The corresponding ultrasound identified a slightly hypoechoic, well-circumscribed nodule of approximately 1 cm (white arrow, (**a**,**b**)), classified as BI-RADS 4. CEM subsequently demonstrated mild mass-type enhancement in the same region (**c**). Ultrasound-guided biopsy revealed grade 2 DCIS. The patient underwent total mastectomy, which confirmed the diagnosis of grade 2 DCIS.

**Figure 4:**
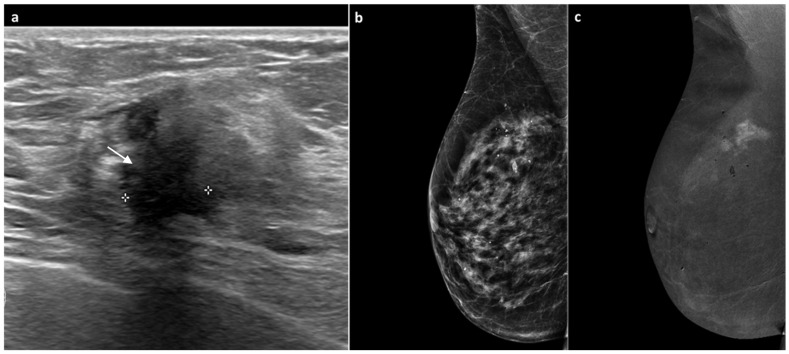
A 77-year-old woman, previously treated for DCIS of the right breast with partial mastectomy 11 years earlier with tenderness at the surgical scar and increased breast consistency. Ultrasound revealed heterogeneous echotexture of the surgical scar with a maximal thickness of 1 cm (white arrow, (**a**)). Mammography showed no significant changes or suspicious findings (BI-RADS 3) (**b**). Subsequent CEM demonstrated a marked, intense, multilobulated mass-type enhancement measuring approximately 1.8 cm (**c**). An ultrasound-guided biopsy diagnosed invasive lobular carcinoma, Luminal B subtype. Total mastectomy confirmed invasive lobular carcinoma with negative axillary lymph nodes.

**Figure 5:**
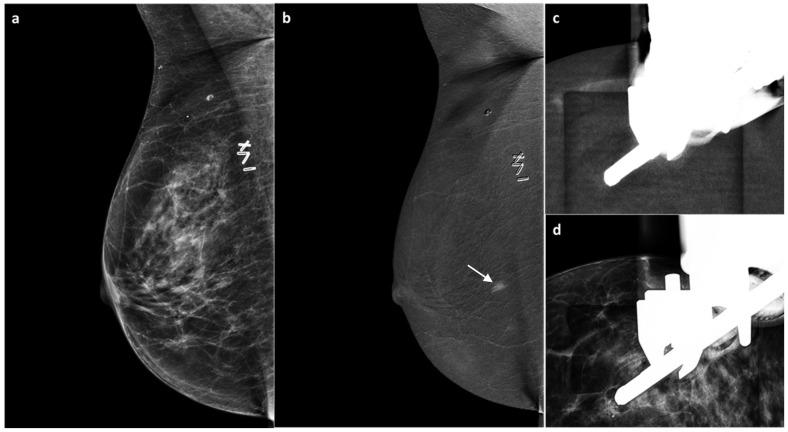
A 55-year-old BRCA-positive woman, previously treated for invasive ductal carcinoma of the right breast with partial mastectomy four years earlier. Follow-up MG revealed no suspicious findings. US demonstrated mild thickening of the surgical scar (BI-RADS 3) (**a**). On subsequent CEM, no enhancement was seen at the surgical scar; however, an area of non-mass contrast enhancement measuring approximately 7 mm was detected in the inner quadrants, with no mammographic or sonographic correlate (white arrow, (**b**)). A CEM-guided biopsy (**c**,**d**) revealed grade 2 invasive ductal carcinoma, Luminal B subtype. Bilateral mastectomy and implant reconstruction were subsequently performed.

**Figure 6:**
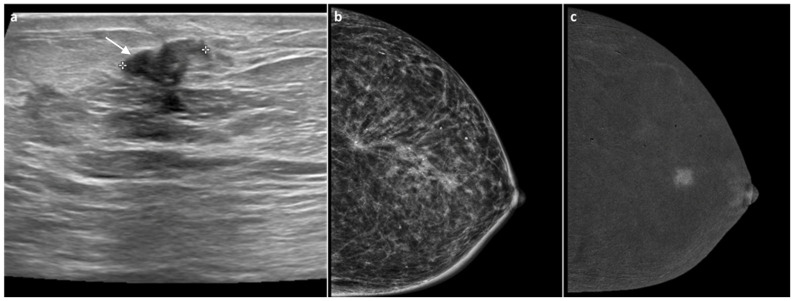
A 90-year-old woman previously treated 10 years earlier for invasive ductal carcinoma of the right breast. The US revealed a nodular lesion measuring approximately 1.2 cm at the site of the surgical scar, with smooth margins (BIRADS 4) (white arrow, (**a**)). MG showed no significant changes, with a diffuse architectural distortion corresponding to the surgical scar, most likely related to postoperative scarring (**b**). CEM demonstrated enhancement of the lesion (**c**). Core biopsy confirmed a diagnosis of grade 2 invasive ductal carcinoma, and a subsequent unilateral mastectomy was performed.

Among the 34 enhancing lesions, 30 (88.2%) were malignant and 4 (11.8%) were benign, while 25 of the 28 non-enhancing lesions (89.3%) were benign and only 3 (10.7%) were malignant. This correlation was statistically significant (*p* < 0.001) confirming that the presence of enhancement on CEM is significantly associated with malignancy (Table 6). ROC analysis demonstrated an excellent diagnostic performance of CEM, with an area under the curve of 0.888 (95% CI: 0.782–0.954, *p* < 0.0001) (Figure 2). Figure 3, Figure 4, Figure 5 and Figure 6 show four representative cases from our study.

## 4. Discussion

Women with a PHBC remain at increased risk for local recurrence or new primary tumors, often diagnosed at a later stage than cancers detected in the general screening population. Conventional imaging techniques, such as MG and US, may be limited in the evaluation of post-surgical breasts due to architectural distortion and scarring. CEM has emerged as a promising imaging tool, providing sensitivity comparable to MRI while offering greater accessibility and lower cost [1,6,11]. It may be particularly useful in evaluating operated breasts, where post-surgical changes and scar tissue often pose diagnostic challenges [16]. However, its precise role in breast cancer follow-up remains under investigation. The present study aimed to evaluate the diagnostic performance of CEM in detecting suspected local recurrence in women with a PHBC.

This prospective study demonstrates that CEM provides excellent diagnostic performance in the surveillance of women with a PHBC who present with suspicious findings on conventional imaging. With a sensitivity of 90.9%, specificity of 86.2%, and an overall accuracy of 88.7%, CEM may help distinguish benign from malignant lesions in the post-surgical breast. A possible explanation for these findings is that CEM provides superior lesion conspicuity by highlighting areas of neoangiogenesis, allowing better differentiation between scar tissue and recurrent tumor compared with conventional imaging modalities. These results are consistent with previous studies and further support the growing body of evidence suggesting that CEM is a valuable tool for the follow-up of operated patients, particularly in cases where conventional imaging is limited by post-surgical architectural distortion or fibrosis. Elder et al. [20] employed CEM as the sole imaging modality for surveillance in women with a PHBC, demonstrating superior sensitivity compared with MG. Their reported diagnostic performance values were lower than ours; however, our cohort included patients presenting with new findings already identified on MG or US as BI-RADS 3–5, reflecting a more clinically targeted population. Furthermore, Gluskin et al. [15] found that among women undergoing screening after breast-conserving surgery, CEM detected more cancers and achieved a higher biopsy positive predictive value compared with mammography. Helal et al. [21] reported diagnostic performance and enhancement values similar to those observed in our study; however, their cohort primarily included recently operated patients. In contrast, our series encompassed women who had undergone surgery more than ten years earlier. Moreover, Berg et al. [17] reported that adding CEM to digital breast tomosynthesis improved early breast cancer detection in women with a PHBC.

Furthermore, the diagnostic accuracy observed in this study further supports the role of CEM as a problem-solving tool in the evaluation of BI-RADS 3 and 4 lesions identified on conventional imaging. By improving lesion characterization, CEM may help reduce unnecessary biopsies and patient anxiety, while ensuring appropriate management of suspicious cases. CEM provided an incremental diagnostic value over conventional imaging, increasing overall accuracy from 83.9% to 88.7%. In BI-RADS 3 malignant lesions, CEM identified all cases and prevented diagnostic delays. For this reason, excluding BI-RADS 3 patients from CEM could delay diagnosis in a relevant subset. These results indicate that CEM adds essential functional information related to vascularity, allowing more accurate differentiation between benign postoperative changes and true recurrence. A slightly higher-than-expected proportion of malignant lesions within the BI-RADS 3 category (9.7%) may be explained by several factors. Post-surgical changes, dense parenchyma, or fibrosis can mimic benign findings and obscure early malignant features, increasing the risk of underestimation. In addition, small or low-grade tumors may present with subtle or non-specific characteristics, leading to an initial “probably benign” classification. Finally, this finding indicates that, in this specific cohort, BI-RADS 3 lesions carry a higher-than-expected malignancy rate and highlights the added value of CEM in refining risk assessment and optimizing clinical management.

Three malignant lesions in our cohort did not exhibit any detectable enhancement on CEM. This finding may be explained by the biological characteristics of these tumors. In particular, low-grade or in situ carcinomas, as well as small invasive lobular carcinomas, are known to have limited angiogenic activity and reduced microvascular density, which may result in minimal or absent uptake of iodinated contrast medium. Moreover, the infiltrative growth pattern typical of lobular carcinoma often leads to a more subtle radiological presentation, with less pronounced disruption of the surrounding parenchymal architecture and lower vascular permeability compared with ductal tumors. Finally, technical factors such as small lesion size, partial volume effects, or background parenchymal enhancement may have further contributed to the lack of visible enhancement. These biological and technical aspects together could account for the false-negative CEM findings observed in these cases. Conversely, a potential source of diagnostic uncertainty arises from lesions that may enhance despite being benign, particularly in the presence of fat necrosis, granulomatous reactions or postoperative inflammatory changes. These conditions can show increased vascular permeability, leading to enhancement that may mimic malignancy. Similarly, background parenchymal enhancement, especially in women with dense breasts, may reduce lesion conspicuity or partially mask true enhancement patterns. In our cohort, four enhancing lesions were benign, and this finding is consistent with the known behavior of inflammatory or necrotic tissue. Despite these potential confounders, enhancement remained strongly associated with malignancy (*p* < 0.001), supporting its diagnostic value while underscoring the importance of integrating enhancement with low-energy images and clinical history to avoid misinterpretation [22].

The data from our study highlight that enhancement observed on recombined CEM images—whether presenting as mass, non-mass, or asymmetric enhancement—is a strong indicator of malignancy. In fact, in our cohort, enhancement was significantly associated with malignant lesions. The authors therefore considered this parameter a key factor in determining the level of suspicion, while always integrating it with findings from the LE images. Moreover, the high interobserver agreement observed in our study (κ = 0.76; *p* < 0.001) underscores the reproducibility of CEM interpretation among experienced readers. This strong association between enhancement on CEM and histopathological malignancy is an important prerequisite for its wider adoption in routine follow-up protocols. Previous studies have reported similar or slightly lower according values [23,24] confirming that CEM provides a consistent and reproducible diagnostic assessment in daily practice. This level of agreement is particularly relevant in the post-surgical setting, where image interpretation is inherently more complex. The exclusion of women with implants and women who have had a mastectomy, while reducing generalizability, were essential due to the technical constraints of post-mastectomy CEM and the impact of implants on contrast behavior and image quality; however, dedicated investigations in these specific populations remain necessary.

In this challenging context, CEM offers both morphological and functional information, aiding the detection of enhancing recurrent lesions and their differentiation from fibrotic or benign post-surgical changes. These advantages make CEM especially valuable for symptomatic patients or those with contraindications to MRI. However, we highlighted that CEM should be regarded as a practical alternative in selected cases, rather than a substitute for MRI.

Despite the encouraging results, several limitations should be acknowledged. This was a monocentric study with a relatively small cohort, potentially limiting generalizability, statistical power, correlation with histopathological profiles and enhancement patterns. The predominance of postmenopausal women may have influenced background enhancement and lesion detectability. The 6-month follow-up used to classify non-enhancing lesions as benign, although practical, may not completely exclude late recurrences. Moreover, the fixed 2 min post-injection timing does not account for kinetic variability that could affect lesion conspicuity. Finally, the ROCs may be influenced by the preselected BI-RADS 3–5 cohort, as the study was specifically designed to assess CEM as a problem-solving tool rather than in a screening setting.

## 5. Conclusions

In conclusion, CEM demonstrated excellent diagnostic accuracy and reproducifbility in the surveillance of patients with a PHBC, reliably distinguishing benign from malignant findings in the operated breast. By offering both morphological and functional information, CEM addresses the limitations of conventional imaging and helps fill the diagnostic gap in this complex clinical setting, providing a valuable and practical tool for more confident follow-up assessment. Given its accessibility and diagnostic reliability, CEM may serve as an alternative to MRI in selected cases, particularly in patients with contraindications to gadolinium-based contrast or limited MRI availability. Further multicentric studies with larger cohorts and long-term follow-up are warranted to confirm these results and to better define the role of CEM within standardized surveillance protocols for women with a history of breast cancer.

## Figures and Tables

**Table 1:** Demographic and clinical characteristics of the study population.

Characteristics	Value
Age (years, mean ± SD)	67.1 ± 12.0
Body mass index (kg/m^2^, mean ± SD)	23.7 ± 8.1
Time from first diagnosis	
<5 years (n, %)	17 (27.4)
5–10 years (n, %)	24 (38.7)
>10 years (n, %)	21 (33.9)
Clinical findings	
Locoregional pain (n, %)	18 (29.0)
Palpable nodule (n, %)	13 (21.0)
Nipple secretion (n, %)	3 (4.8)
Nipple retraction (n, %)	2 (3.2)
Cutaneous retraction (n, %)	3 (4.8)
Palpable adenopathy (n, %)	2 (3.2)
Family history of breast cancer (n, %)	17 (27.4)

Abbreviations—SD: standard deviation.

**Table 2:** Conventional Imaging characteristics of the studied lesions.

Characteristics	Value
BI-RADS	
BI-RADS 3	31 (50.0)
BI-RADS 4	19 (30.6)
BI-RADS 5	12 (19.4)
Side	
Left	40 (65.0)
Right	22 (35.0)
Location of main lesion	
UOQ	28 (45.2)
UIQ	8 (12.9)
LOQ	9 (14.5)
LIQ	11 (17.7)
RA	6 (9.7)

Abbreviations—BI-RADS: Breast Imaging-Reporting and Data System; UOQ: upper outer quadrant; UIQ: upper inner quadrant; LOQ: lower outer quadrant; LIQ: lower inner quadrant; RA: retro-areolar.

**Table 3:** Contrast-enhanced mammography features of the study population.

Characteristics	Value
Glandular pattern	
A	3 (4.8)
B	34 (54.8)
C	17 (27.4)
D	8 (12.9)
Background parenchymal enhancement	
Minimal	32 (51.6)
Mild	18 (29.0)
Moderate	9 (14.5)
Marked	3 (4.8)
Enhancement pattern	
Mass	26 (41.9%)
Non-mass	2 (3.2%)
Asymmetry	6 (9.7%)
No enhancement	28 (45.2%)

**Table 4:** Histopathological and immunophenotypical profile.

Characteristics	Value
**Benign**	7
Fat necrosis	2
Granuloma	5
**Malignant**	33
**Histotype**	
Infiltrating ductal carcinoma	23
Infiltrating lobular carcinoma	6
Ductal carcinoma in situ	4
**Immunophenotype**	
Luminal A	7
Luminal B	11
HER2+ (non-luminal)	2
Triple negative	3
Not typified	10
**Grading**	
G1	17
G2	10
G3	6
**Lymph node localization**	
Negative	31
Positive	2

Abbreviations—HER2: human epidermal growth factor receptor 2.

**Table 5:** Diagnostic performance of CEM and histopathological or follow-up findings.

Parameter	Value
Sensitivity	30/33 (90.9%)
Specificity	25/29 (86.2%)
Positive predictive value	30/34 (88.2%)
Negative predictive value	25/28 (89.3%)
Diagnostic accuracy	55/62 (88.7%)

**Table 6:** Correlation between CEM enhancement and histopathological or follow-up findings.

Enhancement on CEM	Benign Lesions	Malignant Lesions	Total	*p*
**Yes (*n*, %)**	4 (11.8%)	30 (88.2%)	34 (100.0%)	<0.001
**No (*n*, %)**	25 (89.3%)	3 (10.7%)	28 (100.0%)	

Abbreviations—CEM: contrast enhancement mammography.

## Data Availability

The data presented in this study are available on request from the corresponding author due to privacy.
